# Spatial Heterogeneity in Carbon Pools of Young *Betula* sp. Stands on Former Arable Lands in the South of the Moscow Region

**DOI:** 10.3390/plants14152401

**Published:** 2025-08-03

**Authors:** Gulfina G. Frolova, Pavel V. Frolov, Vladimir N. Shanin, Irina V. Priputina

**Affiliations:** 1 Institute of Physicochemical and Biological Problems in Soil Science, Pushchino Scientific Center for Biological Research of the Russian Academy of Sciences, Institutskaya Str., 2, 142290 Pushchino, Russia; frolov@pbcras.ru (P.V.F.); shaninvn@gmail.com (V.N.S.); priputina@pbcras.ru (I.V.P.); 2 Isaev Centre for Forest Ecology and Productivity of the Russian Academy of Sciences, Profsoyuznaya Str. 84/32, 117997 Moscow, Russia

**Keywords:** carbon pools, spatial heterogeneity, *Betula* sp. stands, post-agricultural ecosystems, carbon sequestration, stand density, soil carbon, biomass allocation

## Abstract

This study investigates the spatial heterogeneity of carbon pools in young *Betula* sp. stands on former arable lands in the southern Moscow region, Russia. The findings could be useful for the current estimates and predictions of the carbon balance in such forest ecosystems. The research focuses on understanding the interactions between plant cover and the environment, i.e., how environmental factors such as stand density, tree diameter and height, light conditions, and soil properties affect ecosystem carbon pools. We also studied how heterogeneity in edaphic conditions affects the formation of plant cover, particularly tree regeneration and the development of ground layer vegetation. Field measurements were conducted on a permanent 50 × 50 m sampling plot divided into 5 × 5 m subplots, in order to capture variability in vegetation and soil characteristics. Key findings reveal significant differences in carbon stocks across subplots with varying stand densities and light conditions. This highlights the role of the spatial heterogeneity of soil properties and vegetation cover in carbon sequestration. The study demonstrates the feasibility of indirect estimation of carbon stocks using stand parameters (density, height, and diameter), with results that closely match direct measurements. The total ecosystem carbon stock was estimated at 80.47 t ha^−1^, with the soil contribution exceeding that of living biomass and dead organic matter. This research emphasizes the importance of accounting for spatial heterogeneity in carbon assessments of post-agricultural ecosystems, providing a methodological framework for future studies.

## 1. Introduction

The contribution of forest ecosystems to carbon sequestration is widely recognized [1,2,3]. Estimates of carbon stocks in natural reservoirs of young forests that have been formed on abandoned land are important for understanding the sustainability of ecosystem functioning and characterizing the biological cycle in the early stages of reforestation [4].

The southern part of the Moscow region and other neighboring regions of European Russia are characterized by young birch (*Betula* sp.) forests with admixture of willow (*Salix caprea* L.) that have formed on abandoned arable lands [5,6,7,8,9]. Spatial heterogeneity is often observed in such stands [7,9]. A highly heterogeneous mosaic of openings on aforested former arable lands, which is difficult to analyze using 30-m resolution Landsat satellite images, was observed in the Yaroslavl region [7]. Significant spatial heterogeneity in the horizontal structure of young forest, which is clearly manifested at the local level, has been observed using remote sensing methods on abandoned fields in the Tula region [9]. As demonstrated by the stand density map compiled using multitemporal UAV imagery, density varies from 2 to 6 trees per m^2^, with zones of different densities occurring within 50 m. The authors also draw attention to the patchiness of afforestation in a particular stage of its development. Remote sensing studies of abandoned lands in the Moscow region [8], combined with a historical analysis of fires, showed that, by 2018, only 1% of the studied area had a homogeneous structure and had not been affected by fire. About 10% of the area was characterized by dispersed tree regeneration, 17% was classified as fallow areas with low-density tree cover, and the remaining 72% of the abandoned land had no trees or only a few trees. More than 50% of all heterogeneous areas were affected by fires.

Spatial heterogeneity, which is associated with the spatial structure of plant communities, plays an important role in the distribution of carbon stocks at the biogeocenotic level [10]. This influence is realized through the interaction of combinations of different types of vegetation cover and litterfall, forest floor formation, roots, soil fauna, microbiota, and the hydrothermal regime. Tree location has a significant impact on carbon storage and the distribution of other elements within ecosystem pools [11]. Young trees in post-agricultural forest communities develop a greater mass of fine roots relative to total phytomass compared to older stands. The preferential location of their root systems in the upper organomineral soil layer leads to an increased amount of fine root litterfall income to the former arable horizon [12]. Tree crowns have been shown to affect the total C stock of the forest floor, its fractional composition, and the ratio of C stocks in sub-horizons [13,14,15], as well as increasing soil organic C accumulation [16].

Earlier studies have examined the influence of such aspects of horizontal stand structure as density, canopy structure, and species composition on the distribution of carbon in the forest floor and upper mineral soil layers. These studies were conducted at the local level in middle-aged natural forests [17,18,19,20,21] and young forest plantations with a regular spatial structure [22,23].

Several authors [24,25] have investigated the variability of key carbon cycle indicators in mid- and late-successional temperate forests in North America to improve regional-scale assessment approaches. Data were collected in clusters consisting of four circular plots arranged in a grid within a 1 km^2^ area [26]. The results showed that, across all three forest types studied, stand structure was generally less variable than carbon pools and carbon fluxes. This suggests that traditional sampling procedures, which were developed to assess structural characteristics relevant to timber harvesting, may not adequately characterize carbon pools or fluxes.

Additionally, a study was conducted on the spatial patterns of aboveground net primary production and its components in northern Wisconsin. This study was based on a circular plot sampling method [24], which included 312 circular plots across a rectangular 2 × 3 km area. The results showed that there were significant differences among vegetation types, ranging from 4.5 t ha^−1^ yr^−1^ for treeless wetlands to 7.8 t ha^−1^ yr^−1^ for aspen forests.

To the best of our knowledge, the existing literature contains only a few comprehensive studies that characterize the state of carbon pools in post-agricultural forest ecosystems due to environmental heterogeneity. In [27], the influence of vegetation on the carbon content of the top 5 cm of soil in semi-arid, abandoned ecosystems in Spain was studied; however, the influence of horizontal structure was not analyzed. Considering the spatial heterogeneity of biogeocenoses, a transect and sampling method at reference points was used to identify the factors influencing the formation of vegetation mosaic in a post-agricultural ecosystem undergoing overgrowth with herbaceous-shrub vegetation in the middle taiga subzone (Arkhangelsk region, Russia) [28]. This study demonstrated the variability of soil parameters, such as N and organic C content, at reference points along the transect. Studies of young post-agricultural forests emphasize estimates of phytomass and productivity of trees [29] or grasses [30], but pay little attention to the spatial aspect. Other studies consider the spatial heterogeneity of wood stock distribution when estimating the phytomass of woody plants, but do not consider other carbon pools [31].

Nowadays, there is a lack of comprehensive studies that characterize the relationship between the main carbon pools, including the aboveground (AGM) and belowground (BGM) phytomass of the forest stand and ground layer vegetation (GLV), deadwood, forest floor, and the upper layers of mineral soil, with the environmental heterogeneity formed during the post-agricultural afforestation. Without consideration of such interrelationships, the spatial extrapolation of data obtained through measurements at a local site will result in significant errors when estimating the carbon stocks of both the entire ecosystem and its individual components.

The objective of the study was to analyze the relationships between the complex of environmental factors and the carbon stocks of the main ecosystem C pools of forests formed on former arable lands. The hypotheses tested in this study were as follows: (1) the heterogeneity of the environment (i.e., spatial variation in stand density, mean diameter at breast height (DBH)/mean diameter at root collar (DRC), mean height (H), deadwood stock, and light conditions under the canopy) determines the total ecosystem carbon stocks and the contribution of its individual components; (2) indirect calculation of the carbon stocks of the post-agricultural ecosystem is possible based on data regarding the spatial distribution of stand density, including standing dead trees (SDT), their mean heights, and their mean diameters. These results may improve our understanding of the mechanisms controlling the net carbon balance in forests on abandoned agricultural lands, which is important for predicting carbon sequestration in the transition to a carbon-neutral economy.

## 2. Materials and Methods

### 2.1. Study Area

**Figure 1:**
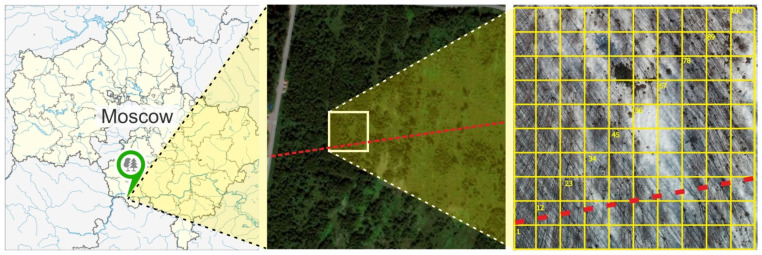
Location and layout of the sampling area. The red dashed line indicates the power transmission line constructed in December 2023, with the associated removal of trees. The PSP is a 50 × 50 m square divided into 100 subplots on the basis of a 5 × 5 m spacing square grid.

The studies were conducted in the Southern Moscow region, on the territory of the ecological-climatic station (ECS) “Pushchino” (54.83377° N, 37.56892° E) [32] (Figure 1). The investigated area is located within the zone of temperate broad-leaved forests [33]. Medium and heavy loamy, light clay with a predominance of silt fraction [34] arable gray soils (Luvisol (Aric)) are formed on loess-like bedrock [32,35].

The reestablishment of forest vegetation has been observed on the plot, which was used in agrochemical experiments for a long time. In the 1970s–1990s, the study area was a part of the experimental field station of the Institute of Soil Science, where various agricultural crops were grown [36]. Prior to being abandoned in 2004, the plot was cultivated as a potato field for several years without the use of fertilizers. As a result of the agricultural technology used, a distinctive microrelief has formed, consisting of almost parallel sections, each about 1 m wide, of furrows and ridges with a height difference of about 0.1–0.2 m. This microrelief remains to this day.

Currently, the study area is covered by a young forest dominated by *Betula* sp. with a heterogeneous spatial distribution of trees. There are ribbon-shaped zones without trees, extending from north to south, as well as irregularly shaped openings lacking trees or containing very few trees. The formation of these structures may have been influenced by fire events. According to a 2018 study [8], this area experienced 3–4 fires between 1985 and 2014. Fire events have been identified in some parts of the area during the herb–grass stage. However, we have no information regarding fires during the development of tree stands, such as burnt trees, fire-induced scars on trees, or charcoals in soil horizons.

**Figure 2:**
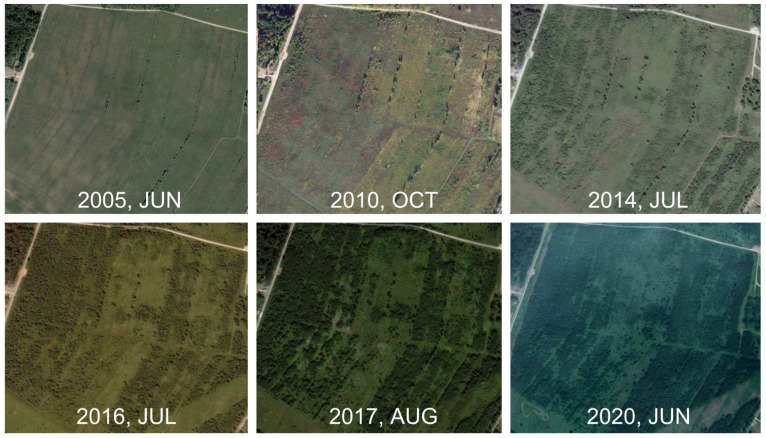
Multitemporal satellite images of the “Pushchino” ECS (Google Earth Pro GIS).

A comprehensive visual chronosequence of afforestation on the site can be traced using Google Earth Pro GIS imagery (Figure 2). In 2005, the fallow area was covered by grass, with the microsite boundaries of the previous agricultural phase remaining relatively clear, being interspersed with sporadic tree growth along these boundaries. The 2010 image indicates that the site had begun to be colonized by trees and shrubs, which were denser along the northern and western boundaries of the fallow field. Here, an edge of mature broadleaf forest was located across the road. The effect of spring burns [37] can also be observed. The 2014 image shows the growth of trees and shrubs across the entire fallow area, except for the central part, which was more slowly colonized by trees. A series of images from 2016, 2017, and 2020 demonstrates the persistent shape and location of the openings.

In July 2023, a permanent sampling plot (PSP) was established in the eastern part of the fallow area, 80 and 150 m from the local roads surrounding the “Pushchino” ECS (Figure 1). The location of the PSP was chosen based on the heterogeneity of the vegetation cover, specifically to present various stand densities. The PSP was designed as a 50 × 50 m square divided into 100 subplots of 5 × 5 m (Figure 1). In December 2023, the stand in the southern part of the PSP (10 × 50 m) was removed during the construction of a power transmission line. Consequently, a 15 m wide buffer zone was established, and all fieldworks in 2024 were conducted solely within the remaining northern part of the PSP (25 × 50 m, 50 subplots). The photographs taken on the PSP can be found in the Supplementary Materials (Figures S1–S3).

### 2.2. Field Studies

Field studies on PSP were performed at different scales at the end of July (Table 1).

To measure the light conditions under the canopy, hemispherical photographs were taken at the center of each subplot using a Canon (Tokyo, Japan) D600 camera equipped with a Sigma (Kanagawa, Japan) AF 4.5/2.8 EX DC HSM Fisheye Canon lens (180° angle of view). The visual axis was directed towards the zenith. The camera was leveled using a bubble level, with the top of the frame oriented towards geographic north, taking into account the magnetic declination, which was calculated for a given location using the World Magnetic Model [38].

A tree survey was conducted in each subplot, during which either the DBH or the DRC, if DBH measurement was not possible, was measured using calipers. Tree height was measured using a Laser Technology, Inc. (Centennial, CO, USA) TruPulse 360B laser rangefinder–inclinometer. Identification of the dominant GLV species was also conducted in each subplot. The projective coverage (hereinafter referred to as “cover”) of GLV was visually estimated on a linear percent scale, along with the sum of the covers of the dominant species.

To determine the AGM, the GLV plants were harvested on a 0.25 m^2^ (0.5 × 0.5 m) area in the center of each subplot at the end of July. Along with living phytomass, litter samples were collected from the same locations within the plot. The litter was primarily composed of the L horizon, which consists of last year’s leaves, branches, and stems of trees, as well as the dead parts of herbaceous plants.

To evaluate the BGM, nine 25 × 25 cm soil monoliths were excavated to a depth of 30 cm. The sampling points were selected to account for the varying density and size characteristics of the stand across the PSP. The local stand basal area within a 1.5 m radius from the sampling point was calculated (due to the root spread radius of *Betula* sp. trees of such size characteristics). During the calculation of local stand basal area, the cross-sectional area of the stems was normalized by the distance from the center of the monolith to each tree within the given radius.

To reveal the relationship between tree size and phytomass, the model trees were sampled outside the PSP near its northern boundary to keep the stand structure unchanged. A total of 24 *Betula* sp. trees were selected, with H greater than 1.3 m and DBH ranging from 1 to 12 cm with a 1 cm step. Within the DBH range from 1 cm to 6 cm, 3 trees were selected for each diameter class, and within the 7–12 cm range, 1 tree was selected for each diameter class. Additionally, seven *Betula* sp. trees with H less than 1.3 m were selected. Sampling of the model trees was carried out in early August. DBH/DRC and H were measured for all trees.

Soil samples were collected from the center of each subplot, from the same locations as the phytomass and litter samples. The samples were collected using a 30 cm Eijkelkamp (Giesbeek, Netherlands) soil sampler. The depth of soil sampling was chosen according to the recommendations of the FAO and the IPCC for estimating soil organic carbon content and stocks [39,40].

### 2.3. Laboratory Studies

Samples of GLV phytomass and litter were separated into fast-decomposable fractions (leaves and aboveground grass litter) and slowly decomposable fractions (branches and tree stems). These fractions were then dried to an absolutely dry mass in paper bags at 105 °C and weighed with an accuracy of 0.1 g.

The soil samples were dried at 60 °C until they reached a constant weight. The dried samples were then ground and sieved through a 0.25 mm sieve. The soil carbon (C) and nitrogen (N) content was determined using a LECO (St Joseph, MI, USA) CHNS-932 analyzer. The bulk density of the mineral horizons was determined using a 100 cm^3^ steel cylinder (Obninsk, Russia). Stocks of C and N for the 0–30 cm layer were then calculated based on the analytically determined C and N concentrations and bulk density.

Tree skeletal roots (with a diameter greater than 2 mm) and fine roots (with a diameter less than or equal to 2 mm), and roots and rhizomes of GLV were manually extracted from the monoliths and sorted into the above-mentioned categories, then dried at 60 °C and weighed. The leaves and live branches of each model tree were dried to constant mass at 105 °C and weighed. The stem was divided into 1 m sections, with the apex (the remaining top part of the stem, measuring less than 1 m) also being included. From the midpoint of each section, disks measuring 10 cm in length were cut. These disks and apices were weighed, then dried at 105 °C until they were completely dry and weighed again. Based on the mass of the 10 cm disks, the mass of the corresponding 1 m section was calculated. The mass of the model tree stem was calculated by summing the masses of the individual 1 m sections and apices. For all model trees, only the AGM was sampled.

### 2.4. Data Processing and Analysis

The images obtained from hemispherical tree canopy photography were binarized by dividing all pixels into two classes: “sky” and “non-sky” based on the intensity in the blue RGB channel. A convolutional neural network based on U-Net technology [41] was used for binarization. These binary images were then used as a mask to calculate the transmittance of both direct and scattered solar radiation [41]. This transmittance was then used to calculate the weighted average global light index (GLI) [42]. The DBH is often used as a predictor of stem mass [43]; however, in young stands with strong inter-tree competition, trees with the same DBH can have remarkably different heights. Consequently, stem volume is a more practical predictor. As the relationship between stem diameter and relative height (i.e., taper curves) is close to linear for young trees, including *Betula* sp. [44], the cone volume equation was used to calculate the stem volume:

(1) $$V_{st}=\frac{1}{3}\times \pi \times {\left(\frac{DRC\;}{2}\right)}^{2}\times H.$$

The DRC was calculated using the equation proposed in [45]:

(2) DRC=1.25 × DBH+1.95.

The DRC was used instead of the DBH in order to involve all trees in the calculations, even those that did not reach a height of 1.3 m. As demonstrated in the relevant publications [43,46,47,48], there is no significant difference between *Betula* sp. and *S. caprea* in stem volume and phytomass allocation among organs. Therefore, the same methodology as used for *Betula* sp. was applied to calculate the stem volume and phytomass distribution for *S. caprea*.

The stem masses were calculated as the product of the stem volumes and wood density, where the latter was estimated at 640 and 415 kg m^−3^ for *Betula* sp. and *S. caprea*, respectively [49,50]. The distribution of phytomass between organs can be described using rank distributions, implemented through power [51,52], and exponential functions [53].

(3) $$m_{i}^{rel}=f\times g^{i},\;i\in 0\ldots 2,$$

where *m_i_* is the mass fraction of organ *i* in the total plant mass (the organ with the maximum phytomass fraction has a rank of 0, the next one has a rank of 1, and so on), f and g are empirical coefficients, with the coefficient f being numerically equal to the mass fraction of the organ with a rank of 0. Taking into account the constant number of phytomass fractions, this dependence can be simplified as follows:

(4) $$m_{i}^{rel}=f\times {\left(\frac{-f+\sqrt{-f\times \left(3\times f-4\right)\;}}{2\times f}\right)}^{i},\;i\in 0\ldots 2.$$

While the distributions of this kind have been shown to be quite stable for mature trees, the plasticity of the ratio of phytomass fractions was more pronounced in young trees. This resulted in higher variability in the coefficients of these equations. Data on phytomass fractioning in model trees were analyzed to determine the dependence of the coefficient *f* on DRC. The following dependence was obtained using nonlinear regression:

(5) $$f=\frac{0.89}{1+2.03\times e^{-1.34\times DRC}}.$$

Calculating the absolute ($m_{st}$) and relative (portion of total phytomass, $m_{st}^{rel}$) stem masses allowed the determination of the entire tree phytomass using the following equation:

(6) $$m=\frac{m_{st}}{m_{st}^{rel}}.$$

The above-described phytomass calculation algorithm was applied to each tree recorded on the PSP. The phytomass calculated for trees and GLV plants was converted to C stocks, taking into account the species-specific C concentrations in different organs.

The following methodology was applied to determine whether C stocks in the main phytomass and mortmass pools could be derived from stand density (with SDT), DBH, and H averaged per subplot. The AGM and BGM of the stand, as well as the GLV, WD, litter, and soil C stocks, were calculated using the previously identified relationships of these parameters to the average per-subplot stand densities (with SDT), mean DBH, and H. These parameters were calculated for those subplots for which measured values were available. Stand and GLV phytomass were converted to C stock using the above-described methodology.

### 2.5. Statistical Analysis

The K-means clustering method was used to differentiate all 100 subplots within the PSP into three classes according to a set of metrics, including mean DRC and H values, GLI, and stand density (calculated by accounting for the amount of SDT). Principal component analysis (PCA) was performed in the R statistical programming environment [54] to identify the primary factors contributing to variation between subplot classes. All measured subplot-level parameters were used as factors for indirect ordination. These analyses were used to identify functionally distinct sub-communities, thereby facilitating spatial interpolation. The interpretation of the ordination axes was based on the values of correlation coefficients with the considered factors.

The imputation of data on the AGM of GLV and the content of C and N in soil for the part of PSP subjected to logging was carried out using the following methodology. A correlation analysis was performed using Spearman’s correlation coefficient to identify the factors exhibiting the strongest relationship with the parameters to be recovered. These factors were then used as predictors to obtain the functional dependencies of the recovered parameters via nonlinear regression. Local stand basal area was used as a predictor to recover data on the BGM of woody and herbaceous vegetation.

To compute the confidence interval for all variables used for the parameterization of functions applied in data imputation, a confidence interval construction approach based on the bias-corrected and accelerated (BCA) method was employed [55,56]. This method accounts for non-normality, bias, and non-constant standard error of the bootstrap distribution, which is often asymmetric. This metric was chosen because of the small or modest sample size and the violation of the asymptotic normality assumption, as in such cases, both parametric asymptotic intervals and nonparametric intervals are unreliable.

To calculate the confidence interval of the predicted values, we used a Monte-Carlo approach to nonlinear error propagation: (1) the mean value and standard deviation of all predictor variables *m* and the variance–covariance matrix of the sums of the fit parameters *β* were used as inputs; (2) for each variable *m*, *n* samples were created from a multivariate normal distribution using the variance–covariance matrix: *x_m,n_*~*N* (*μ*, *∑*); (3) the function *y_n_*=*f* (*x_m,n_*, *β*) was evaluated for each simulated variable; (4) the quantile-based confidence intervals on the vector *y_n_* were calculated.

The same technique was used to compare the parameters calculated during data imputation for the measured and reconstructed parts of PSP. Two statistical hypotheses were tested: (1) the mean values of the analyzed parameters for the measured and reconstructed parts of PSP do not differ; (2) the distributions of the analyzed parameters for the measured and reconstructed parts of PSP do not differ. As none of the analyzed parameters followed a normal distribution (Shapiro–Wilk test at a significance level of 0.05), the non-parametric Wilcoxon’s test was used to test the hypothesis of equality of mean values of the analyzed parameters. The distributions of the above parameters were compared using the exact two-sample Kolmogorov–Smirnov test.

We used the non-parametric Kruskal–Wallis test to compare trees located in subplots of classes that differed in terms of median H and DRC values. This was because the DRC and H values did not follow a normal distribution, and the sample size for Class 3 (n = 5) was too small to apply the central limit theorem. The Kruskal–Wallis test enabled us to determine whether there are any differences between at least two groups. However, it does not provide information about which specific groups differ from each other. Therefore, Dunn’s test was used for a post-hoc analysis of pair-wise group comparison, with a significance level of 0.05 applied.

**Figure 3:**
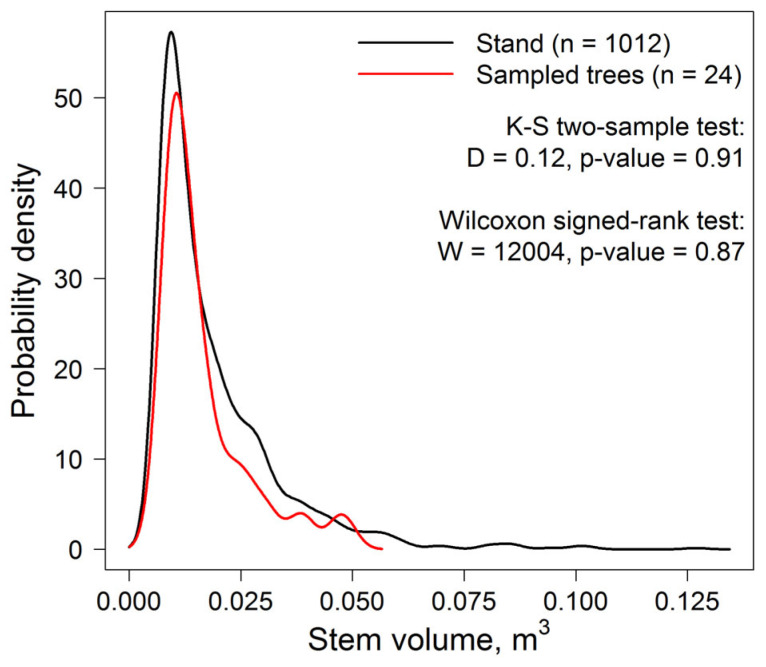
Comparison of stem volume distributions for sampled model trees and for the stand on the PSP.

To verify the representativeness of the sampled model trees in relation to the stand on the PSP, the distributions of stem volumes were compared. The results of the Kolmogorov–Smirnov test showed that these two distributions were not statistically significantly different. Applying the Wilcoxon signed-rank criterion to compare the median values also showed no statistically significant differences between the samples (Figure 3).

The stem masses of the model trees were measured to validate the mass calculation using Equation (1) described above. The validation procedure involved plotting pairs of emission values in “simulated–measured” coordinates and approximating them with the linear regression model *y* = *s* × *x*. The coefficient of determination (R^2^) was used to quantify random deviations, while the slope coefficient (s) represented systematic deviations.

To analyze the possibility of application of the data imputation method described above to averaged data, the differences in C stocks obtained during field studies (“measured”) and those calculated based on averaged stand characteristics (density, mean H and DBH) in subplots (“simulated”) were assessed using the non-parametric permutation method [57]. The null hypothesis proposed that there were no differences between the measured and simulated C stocks.

The corresponding C stocks in the subplots (in an amount equal to the number of subplots in the compared original plots) were randomly selected with return (9999 repetitions). Thus, a total of 9999 pairs of virtual plots were obtained. For each pair of virtual plots, the absolute value of the difference in median values of C stock was calculated. The absolute value of the difference in the median values was also calculated for the “experimental–simulated” pair of plots.

The obtained set of absolute values of differences between medians (including those for the “experimental–simulated” pair of plots) was then sorted in ascending order, and the ordinal number (rank) of the value corresponding to the “experimental–simulated” pair of plots was determined. The P-value was calculated as follows:

(7) $$P=1-\frac{R_{m}^{o-s}}{N},$$

where $R_{m}^{o-s}$ is the rank of the absolute values of the differences between the median values of the plot pairs, and *N* is the total number of plot pairs, including the “experimental–simulated” pair. The null hypothesis was rejected at *p*-value < 0.1.

A comparison of median values of C stock in the main ecosystem pools, calculated for subplots belonging to different classes, was carried out with the same technique as for the comparison of DBH and H belonging to different classes.

## 3. Results and Discussion

### 3.1. Species Composition of Vegetation on PSP

According to the obtained data, the stand on PSP is predominantly composed of *Betula* sp., accounting for 82.3% of the total number of stems, followed by clumps of *S. caprea* (17.1%), and single *Pinus sylvestris* L. trees (0.6%). These tree species are typical for the initial stage of forest succession on abandoned lands in the forest zone of European Russia [58,59]. Young tree regeneration up to 0.5 m in height is more diverse, comprising a variety of species of broadleaf forests. These include *Acer platanoides* L., *Quercus robur* L., *Populus tremula* L., *Acer negundo* L., *Tilia cordata* Mill., *Pinus sylvestris* L., *Sorbus aucuparia* L., *Betula* sp., *Physocarpus opulifolius* (L.) Maxim., *Picea abies* (L.) H. Karst., and *Ulmus glabra* Huds.

The GLV was represented by 44 species of vascular plants (mosses have very little biomass and were not considered in this study). The dominant species were *Solidago canadensis* L., *Aegopodium podagraria* L., *Carex sylvatica* Huds., *Calamagrostis epigejos* (L.) Roth, and *Fragaria vesca* L. A similar composition of GLV dominants presented using the light-demanding species of open habitats was shown in [8,29].

### 3.2. Analysis of Spatial Variation in Vegetation and Soil Across the PSP

The density of *Betula* sp. on the PSP was estimated at 4048 ha^−1^, while that of *S. caprea* was 1936 ha^−1^ (see Table 2 for further details). The total density of about 6000 ha^−1^ was close to the maximum values presented in [9] for similar birch reforestation of the same age in the neighboring Tula region.

**Figure 4:**
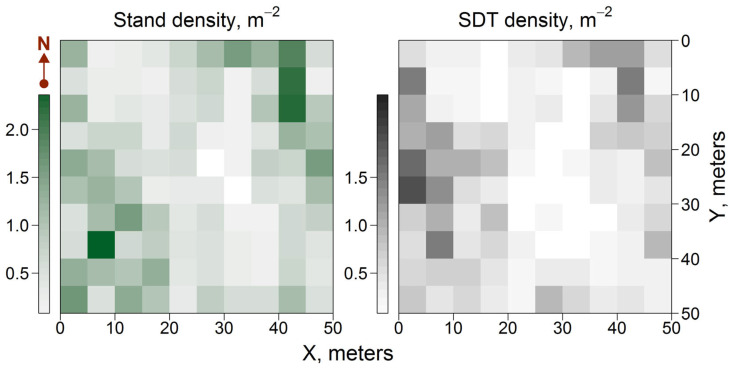
Spatial distribution of the stand and SDT density on the PSP.

The survey of trees within the PSP revealed variations in stand density across the subplots. The proportion of SDT was approximately 30% of the total number of stems. The greatest amount of SDT (4800–14,800 ha^−1^) was observed in subplots with denser stands (12,000–21,000 ha^−1^), which were associated with trees dying off (known as self-thinning) in conditions of higher competition for environmental resources, mostly light (Figure 4). Another possible cause of tree mortality in the PSP could be the dry conditions of some years during the reforestation period; however, the effect of this factor on a local scale, as in this study, should be consistent across the entire PSP. It is unlikely that dry conditions could explain the differentiation of CWD between subplots, as opposed to competition for resources between neighboring trees, which is greater in denser stands.

**Figure 5:**
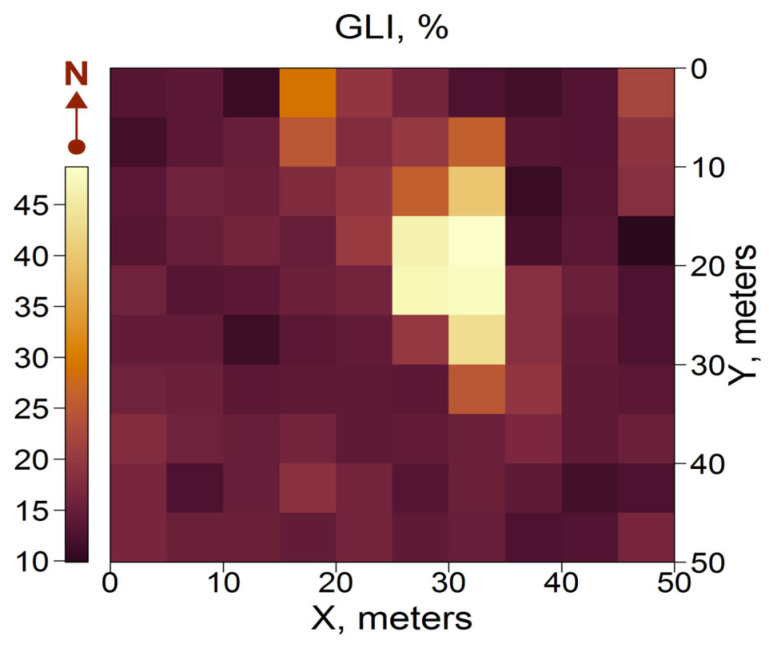
Spatial variation in light conditions under the canopy (GLI).

Stand density (primarily living trees) was found to be a determining factor in the spatial heterogeneity of light conditions under the canopy (Figure 5). GLI exhibited significant variability within the PSP, ranging from 10% to 45% (mean ± SD = 17.3 ± 7.8). A slight discrepancy was observed between the light conditions and the corresponding stand density in each subplot, because a considerable portion of direct solar radiation comes from the lateral directions due to the solar movement. Consequently, the light conditions in a given subplot depend significantly on the stand density in subplots located to the south of it, and, to a lesser extent, to the east and west.

The spatial distribution of light under the canopy depends mainly on its structure. This is often the main limiting factor for GLV species, affecting the abundance and biomass production of these plants.

**Figure 6:**
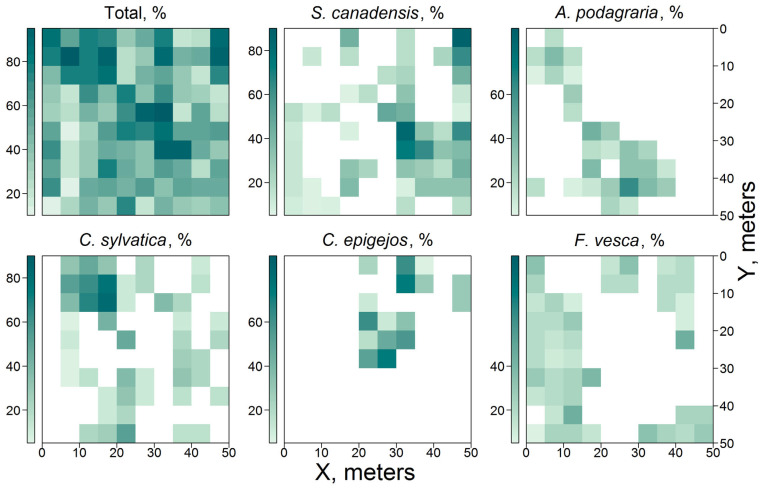
Spatial variation of the cover of GLV (total and for individual dominant species).

The distribution of the dominant GLV species within the PSP is shown in Figure 6. Total GLV cover was found to have a weak correlation with light conditions under the canopy. The highly invasive, light-demanding species *S. canadensis* formed monodominant communities in the canopy opening and in the northeastern corner of the PSP. These communities were characterized by GLI values between 25% and 45%. Additionally, in some subplots within the canopy opening, *C. epigejos* was observed to co-dominate with *S. canadensis*, with total cover reaching 70%. In subplots located in the north-western corner of the PSP with a GLI index <25%, the total cover of *C*. *sylvatica* and *A. podagraria* reached 95%. A further 39 species of vascular plants occurred sporadically within the PSP, with cover up to 10%. Consequently, the vascular plants in the PSP formed areas with high projective coverage, irrespective of light conditions, which can be explained by their spatial distribution in the previous post-agricultural stage of meadow succession. The particular qualities of these species enable them to hold onto occupied habitats for a longer time after changes in ecological conditions [60,61,62]. In addition to the main factors that determine the boundaries of ecological niches and consequently the formation of plant mosaics within a community, random factors such as seed dispersal and subsequent germination, as well as the peculiarities of the meso- and microrelief, can be extremely important at the initial stages of forest succession. The heterogeneous and changing over time environment of young forests can produce a variety of mosaic combinations, which lay the basis for maintaining the most stable species combinations at subsequent stages of forest ecosystem development.

**Figure 7:**
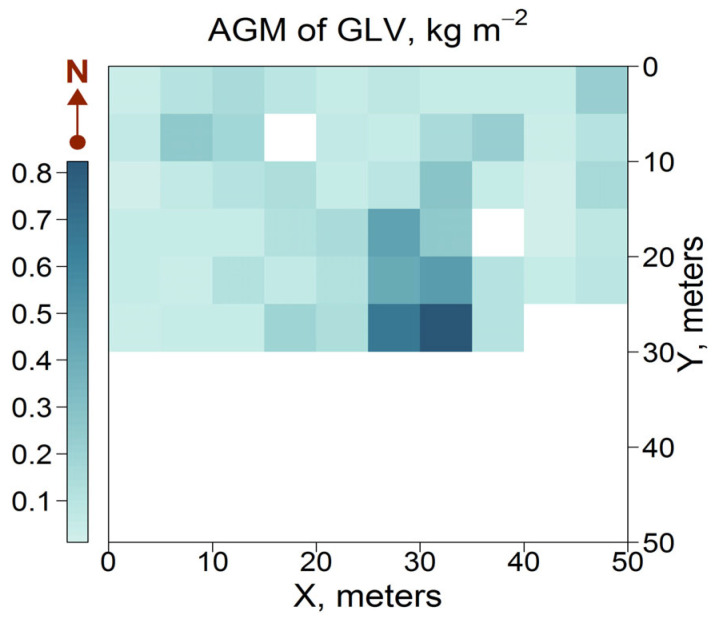
Spatial distribution of AGM of GLV plants. Here and below: the AGM of GLV was not recorded for the southern part of PSP, as it was destroyed during the construction of the power transmission line in December 2023.

As shown in Figure 7, the spatial variation in AGM of GLV was positively correlated with GLI (contrasting with GLV cover), with a single zone of high values. In subplots with the highest AGM of GLV (0.6–0.8 kg m^−2^), predominantly formed by *S. canadensis* in the canopy opening, GLI was recorded at 40−45%. In subplots with minimal AGM of GLV (0.1–0.2 kg m^−2^), GLI was found to be 10–15%. All above-mentioned GLV dominants occurred in these subplots; however, due to a suboptimal combination of environmental factors, these species did not achieve high phytomass values. In the north-western part of the PSP, where the total cover of *A. podagraria* and *C. sylvatica* was high (90%), the phytomass was minimal (0.2–0.3 kg m^−2^). This phenomenon can be attributed to the size characteristics of the dominant species. The height of *A. podagraria* and *C. sylvatica* can reach up to 100 cm, whereas *S. canadensis* can grow to 200 cm in height. This suggests that the phytomass of these species may vary despite similar values of cover. A weak or nonlinear relationship between these parameters has previously been noted in other vascular plants [63,64].

The study of the heterogeneous structure of vegetation cover in post-agricultural ecosystems, which consist of zones with woody vegetation and open spaces with different GLV dominants, including *S. canadensis*, is relevant in the context of analyzing the potential of C accumulation in post-agricultural ecosystems. The long-term persistence of the location and size of canopy openings suggests that zones occupied by herbaceous vegetation (*C. epigejos* and *S. canadensis*) prevent the renewal of woody vegetation over quite a long period. This heterogeneity can lead to the establishment of distinct ecological, cenotic, and edaphic conditions. These conditions subsequently influence the structure of phytomass and mortmass, which, in turn, affects the heterogeneity of C stocks.

**Figure 8:**
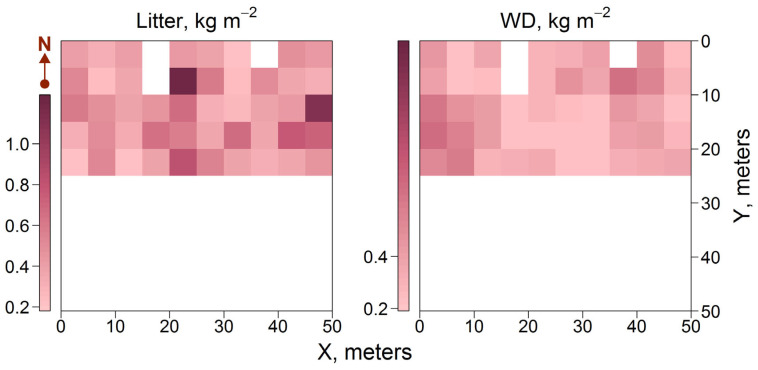
Spatial distributions of fast-decomposable fractions of litter and WD.

The amounts of litter and woody debris (WD) that accumulated on the PSP were within the range of 0.13–1.34 kg m^−2^ and 0.01–0.53 kg m^−2^, respectively. The spatial distribution of WD within the PSP was determined by the presence and number of trees in different subplots. The highest WD stocks were found in subplots with dense stands (Figure 8). The distribution of fast-decomposable aboveground litter fractions, such as foliage and AGM of GLV, was influenced by both stand structure and the distribution of GLV plants, thus complicating the spatial pattern.

**Figure 9:**
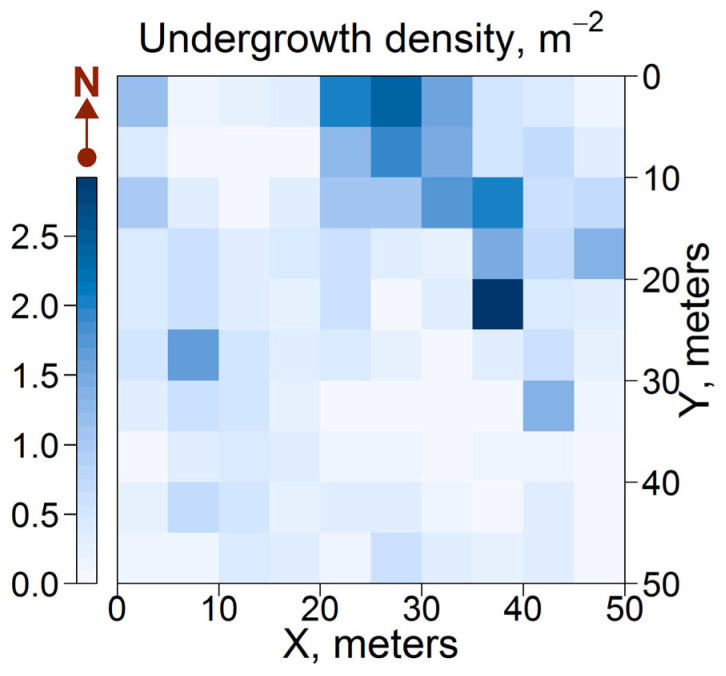
Distribution of tree undergrowth density.

The spatial distribution of undergrowth (defined in this study as young trees up to 0.5 m in height) showed a weak correlation with GLI. Woody regeneration occurred more frequently in subplots located along the northern and northeastern boundaries of the central canopy opening, where the density of seedlings was 1–3 m^−2^ (Figure 9). These subplots were characterized by a GLI of 10–30%, a low AGM of GLV (0.1–0.3 kg m^−2^), and moderate levels of GLV cover (30–70%). This combination of conditions was likely optimal for seed germination and establishment of seedlings of broad-leaved, small-leaved, and coniferous tree species. Other studies [65] have also demonstrated that the seedlings of the majority of tree species in the European part of Russia have high shade tolerance, which is subsequently followed by an increase in light demand during ontogenesis.

**Figure 10:**
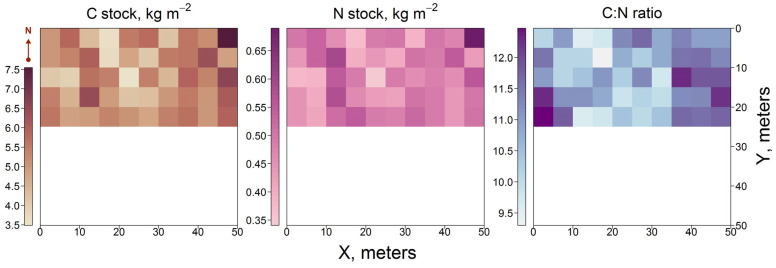
Distribution of C and N stocks and C:N ratio in the soil layer (0–30 cm) at the PSP.

Analysis of soil samples collected at the PSP revealed minimal variation in the bulk density of the top soil layer (0–30 cm), which was found to be 1465.4 ± 3.7 kg m^−3^, with no spatial variation detected. A more complicated spatial distribution was obtained for soil pools of C and N. This higher variability in comparison with other soil characteristics could be due to the distribution of fine roots of trees across the plot, given the higher concentrations of C and N in fine roots litter. The C stock in the 0–30 cm layer was estimated within 3.5–7.5 kg m^−2^, and the N stock within 0.35–0.65 kg m^−2^ (Figure 10). For comparison, the average values of C and N pools in the same soil under agricultural rotations were estimated at 4.7 kg m^−2^ and 0.4 kg m^−2^, respectively [58]. Thus, alongside the general trend of increasing SOC stocks during post-agricultural reforestation, C pools in former arable soils have remained the same or even decreased in some subplots of the PSP. Similar results showing high spatial variability of SOC stocks in abandoned lands have been presented in [28,59].

The C:N ratio, with values ranging from 9.5 to 11.0, was observed in the soil of subplots belonging to canopy openings and areas with sparse stands, where the contribution of GLV litter with higher nitrogen content was greater. C:N values of 11.0–12.0 were observed in subplots with denser stands, where litter is mostly formed by tree leaves, which have a lower N content than herbs. Therefore, the observed spatial distribution of the C:N ratio in the upper soil layer can be explained by the quantity and chemical properties of the litterfall entering the soil in different subplots.

**Figure 11:**
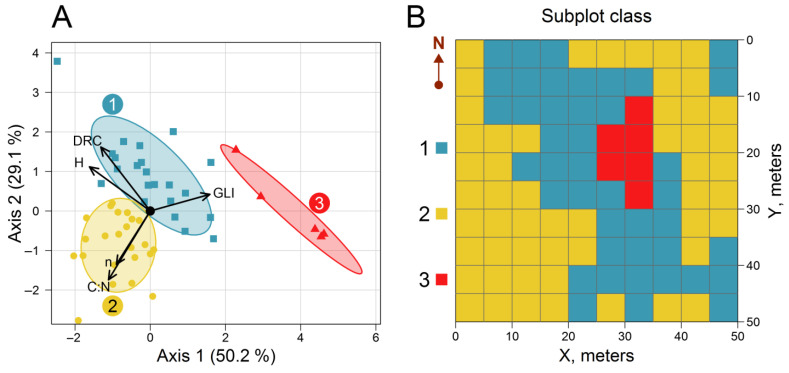
Results of the ordination of subplots using the principal component analysis (PCA) method (**A**) and spatial arrangement of subplots attributed to different classes (**B**).

Cluster analysis was performed using the aforementioned parameters, which were available for all 100 subplots. As a result, the subplots were divided into three classes containing 42, 52, and 6 items, respectively. Principal component analysis (PCA) showed that the first two axes accounted for 79.3% of the variation between subplot classes. The main factors contributing to variation between subplots were the mean DRC of living trees, GLI, and the C:N ratio of the 0–30 cm soil layer. GLI made the most significant contribution to Axis 1, while Axis 2 was predominantly influenced by DRC and the C:N ratio, the effect of which was multidirectional (Figure 11A).

Class 1 encompasses subplots characterized by high DRC values and more fertile soils (the C:N ratio was used as a measure of soil fertility). Class 2 includes subplots with poorer soils and lower DRC and GLI than Class 1 subplots. Subplots belonging to Class 3 are characterized by high GLI values and a wide range of DRC and C:N ratio values (Figure 11B). PCA analysis revealed that an increase in stand density is accompanied by a rise in the soil C:N ratio. This phenomenon may be attributed to the high proportion of tree foliage and fine roots in the litterfall composition, alongside the low proportion of nitrogen-rich GLV litterfall.

Data obtained from the PCA analysis were used for data imputation to prevent the selection of predictors with unidirectional influence and thereby avoid multicollinearity and overfitting of the regression model.

### 3.3. Individual Tree Characteristics

**Figure 12:**
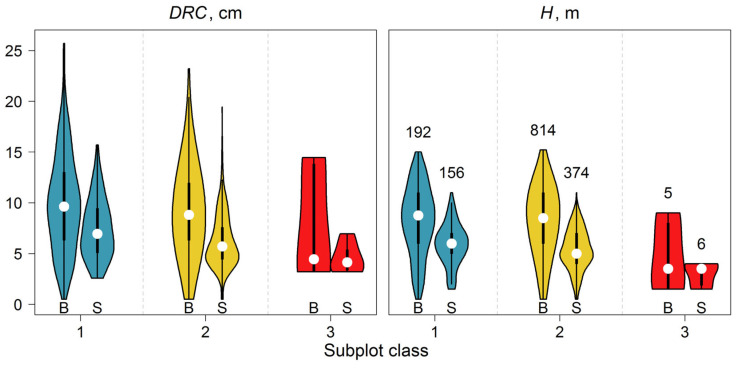
Distributions of DRC and H values in subplots of different classes. Numbers denote the number of trees in each group (B—*Betula* sp., S—*S. caprea*).

To obtain more precise estimates of stand phytomass and, consequently, its C stock, this parameter was calculated on a tree-by-tree basis. The DRC of *Betula* sp. trees varied between 0.3 and 26.0 cm (median value at 8.5 cm) and exhibited a left-skewed distribution (Figure 12), whereas the height distribution was symmetric, with values ranging between 0.5 and 15.2 m and a median value of 8.5 m. This may be because trees under competitive pressure predominantly exhibited height growth, while only the most successful individuals had the opportunity to increase in diameter.

The median values of the H and DRC of *Betula* sp. and *S. caprea*., which are located in different subplot classes on PSP, are shown in Table 3.

Subplots belonging to Class 3 were characterized by a low stand density (0.033 m^−2^) compared to subplots of Classes 1 and 2, which had densities of 0.18 and 0.63 m^−2^, respectively. This resulted in reduced competition for resources in these subplots, consequently leading to lower tree heights at similar diameter values. These disparities suggest that the peculiarities of the stand spatial structure during its formation (over a period of 20 years) did not result in different size characteristics between trees growing in subplots of medium and high stand density. However, distinctions in the size of trees growing in open spaces are evident. It is important to note that the statistical power of the criterion used is low due to the small sample size (n = 5) in Class 3.

**Figure 13:**
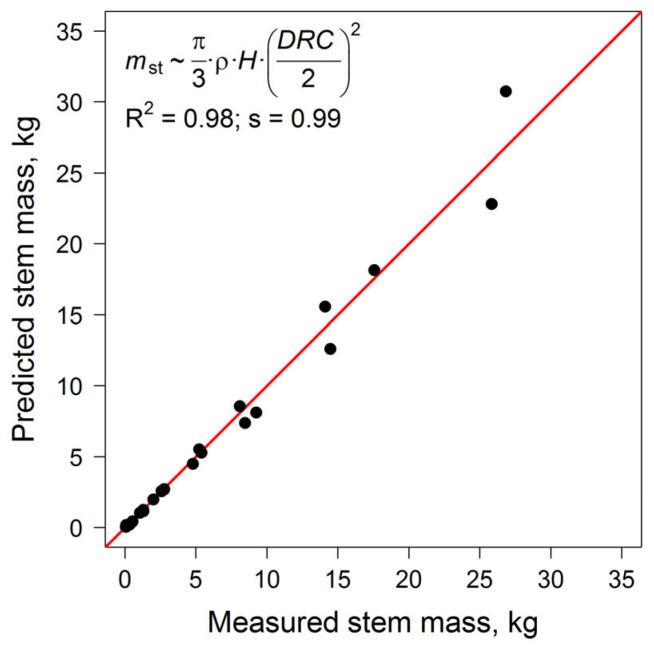
The validation of the stem mass calculation algorithm by comparing the measured and predicted values for trees with the same size characteristics. The coefficient of determination (R^2^) quantifies random deviations, and the slope coefficient (s) quantifies systematic deviations. The red line corresponds to the identity line (1:1).

A comparison of the measured and calculated stem masses showed good agreement (Figure 13).

**Figure 14:**
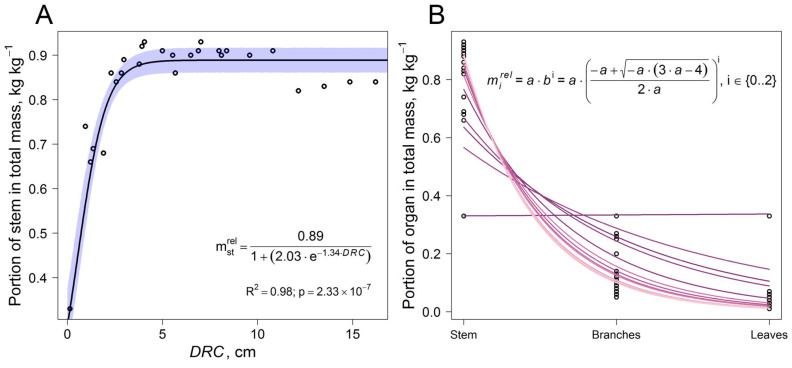
Dependence of *Betula* sp. stem mass portion in total tree phytomass on DRC (**A**) and allocation of *Betula* sp. phytomass among organs as a function of stem mass portion (**B**). Here and below: violet area corresponds to the 95% confidence interval.

In addition to stem mass, the mass of the other fractions (branches and leaves) was also determined. Given that the proportion of stem mass in total phytomass increases with tree growth and can vary significantly, the dependence on DRC was obtained (Figure 14). To calculate the proportion of the other fractions in total phytomass, a dependence based on rank distributions using an exponential function was used [53].

The observed structural relationships indicated a stable regularity in biomass partitioning among organs, associated with their functional role. As is known, the most fundamental processes in plant metabolism are photosynthesis and respiration. Light interception, as well as water and nutrient uptake and transport, are crucial processes that affect the growth, development, and survival of plants. These functions greatly determine biomass allocation to different organs. Compared to mature trees, young trees allocate a larger proportion of their biomass to assimilating organs, accounting for up to 33% of their AGM.

### 3.4. Data Imputation

**Figure 15:**
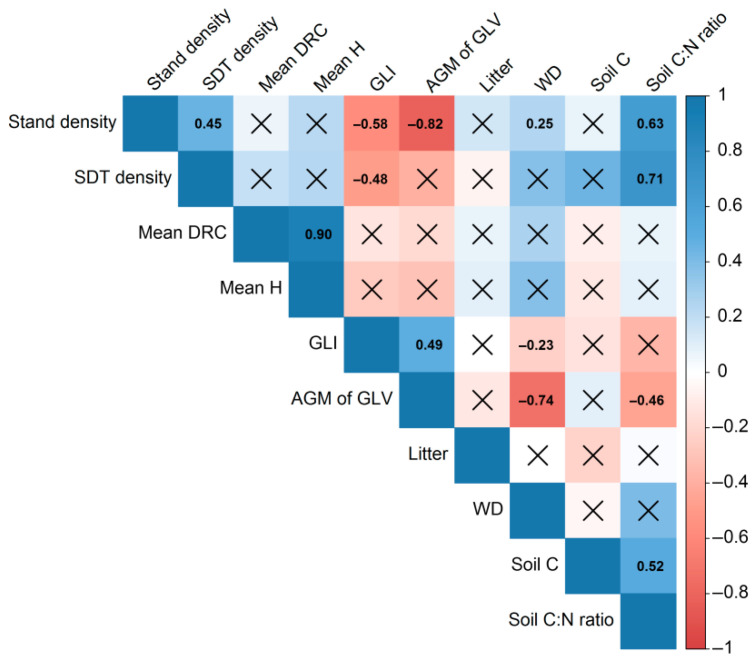
Correlogram of correlations between the parameters measured on the PSP. The numbers indicate the statistically significant values of the Spearman’s correlation coefficients (at a significance level of 0.001).

Correlation analysis of the entire dataset enabled the identification of parameters exhibiting the strongest correlation with those requiring data imputation (Figure 15).

Following a comprehensive analysis of the available data, the following parameters were selected as predictors: stand density for AGM of GLV and stand density (with SDT) for the soil C:N ratio. For WD stock and soil C stock, the correlation with the parameters determined for all subplots was either significantly weaker than that with one of the parameters to be recovered, or statistically insignificant. Therefore, AGM of GLV and soil C:N ratio were selected as predictors for WD stock and soil C stock, respectively. No significant correlation was found between litter stock and other measured parameters. There was also no relationship with the subplot classes defined in this study. Consequently, to recover the missing data, a sample was generated with the same statistical characteristics (mean, variance, and distribution) as the sample of measured values.

**Figure 16:**
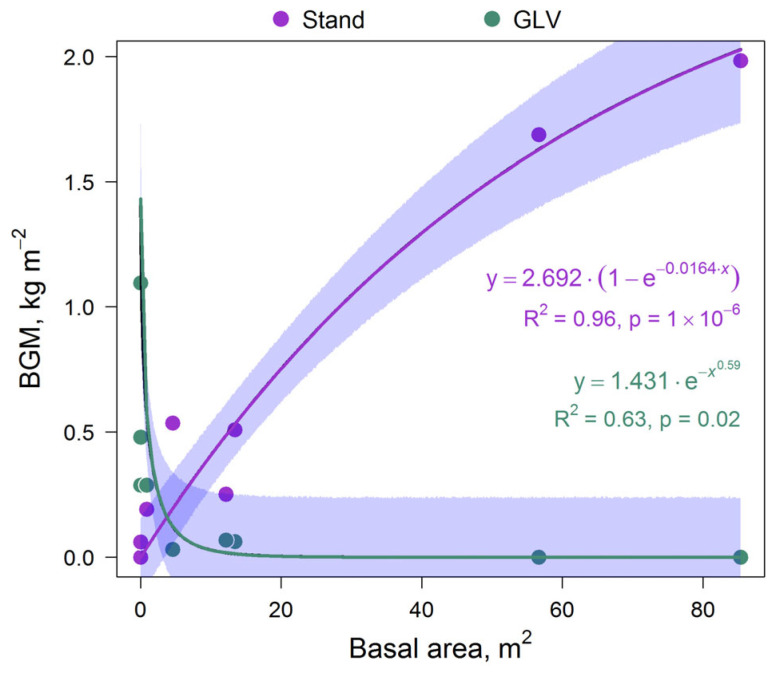
Relationships between the BGMs of stand and GLV and stand basal area.

We found that the mass of *Betula* sp. roots increased as stand basal area increased, while the BGM of GLV decreased (Figure 16). In both cases, this dependence could be described by an exponential function. The mean values comprised 0.58 and 0.26 kg m^−2^, with a maximum of 1.98 and 1.09 kg m^−2^ for BGM of *Betula* sp. and GLV, respectively.

**Figure 17:**
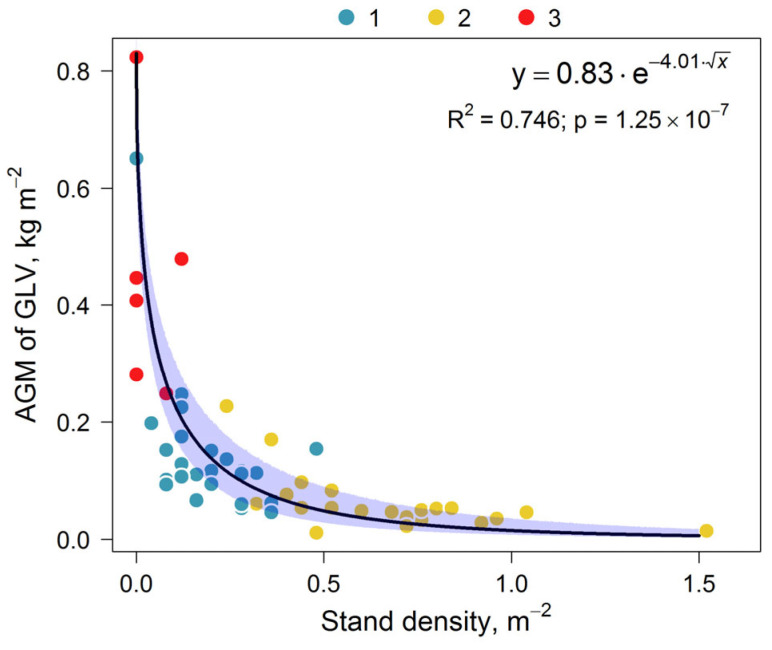
Dependence of AGM of GLV on stand density. Color indicates subplots of different classes (similar to Figure 11).

The dependence of the AGM of GLV on stand density for subplots of different classes, as well as the dependence of the BGM on stand basal area, can be described using a decreasing function (Figure 17). This can be attributed not only to the stand effect on the light available for GLV, but also on its mineral nutrition.

As shown in Figure 17, the relationship demonstrated the intensity of the edificatory effects of stand density (as a manifestation of horizontal stand structure) on the AGM of GLV. Trees in subplots of Class 3 may already be losing their edificatory role as these subplots have abundant herbaceous plants (*C. epigejos*, *S. canadensis*). A similar relationship was observed in studies of abandoned agricultural lands in the Krasnoyarsk Territory (Russia), where an increase in *P. sylvestris* density resulted in a significant reduction in biodiversity, cover, and AGM of GLV [30].

**Figure 18:**
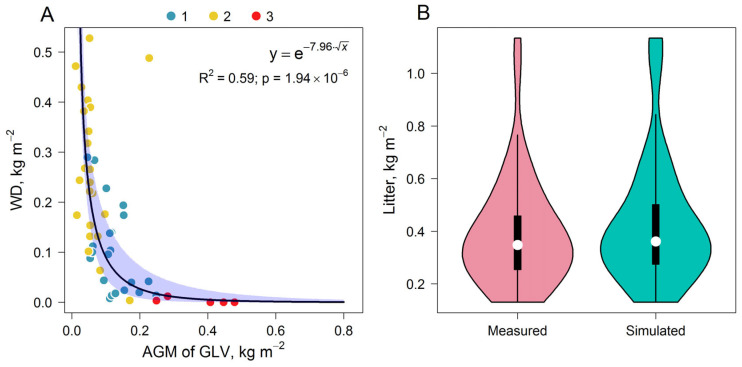
Dependence of WD mass on AGM of GLV (**A**) and distribution of litter stocks measured at the PSP and simulated (**B**).

The relationship between the mass of WD and the AGM of GLV can be described by a negative exponential function, as can the relationship between the AGM of GLV and stand density (Figure 18A). The mean litter stock was 0.39 kg m^−2^, and the distribution is left-skewed (Figure 18B).

The presence of WD in the litter indicated that woody vegetation grew in this area in the past. Low quantities or an absence of WD were characteristic of all Class 3 subplots, some Class 1 subplots, and one Class 2 subplot. This suggests that Class 3 subplots may initially have been colonized primarily by rhizomatous grasses (*C. epigejos* and *S. canadensis*). In Class 1 subplots, colonization by woody vegetation likely occurred non-uniformly over time and space, altering the glade boundary pattern. Class 2 subplots were probably colonized by trees in the initial stage of afforestation and have remained in these areas ever since.

**Figure 19:**
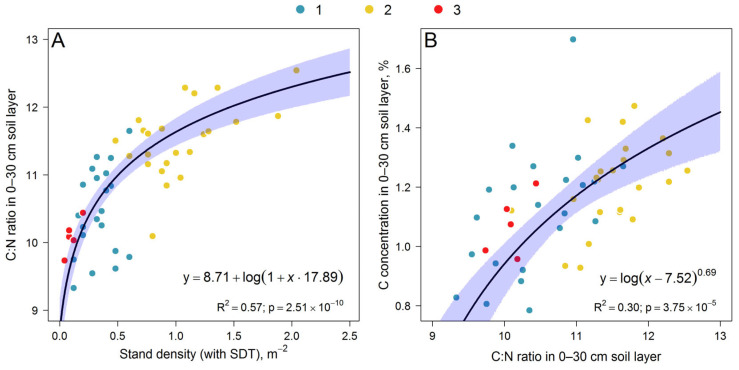
Dependence of C:N ratio in 0–30 cm soil layer on stand density (with SDT) (**A**) and dependence of soil C concentration on soil C:N (**B**).

The dynamics of C stocks in the upper soil horizons exhibited an inertial delay with respect to the amount and qualitative composition of incoming litterfall [66]. In this regard, the actual C:N values were influenced not only by the stand structure and, consequently, the GLV structure at the time of measurement, but also by the structure that contributed to litter formation one or more growing seasons ago. The strongest relationship was consequently found between the soil C:N ratio in the 0–30 cm layer and the total stand density (with SDT) (Figure 19A).

Trees that died before the survey had produced above- and below-ground litterfall annually during their lifetime, and this C flux was then incorporated into the SOC formation process. Changes in stand density and forest boundaries, which occur naturally in young forests on abandoned lands, may be a factor leveling differences in soil parameters between neighboring areas with different vegetation. This phenomenon must be considered when designing research and soil sampling strategies for early successional forests on abandoned lands.

Subplots with higher stand densities were observed to have higher soil C:N ratios. Consequently, subplots with higher C:N ratios have higher C stocks (Figure 19B). This phenomenon can be attributed to the higher quantitative contribution of woody vegetation on PSP to the input of litterfall compared to GLV plants. However, due to the higher N concentration in GLV litter fall, subplots with lower stand density were found to have greater soil richness (in terms of C:N ratio) and, consequently, a greater contribution from GLV plants to litter fall formation.

The equations used to calculate the parameters are given in Table 4.

The analysis showed that there were no differences in the mean values of the parameters calculated during data imputation for the measured and reconstructed parts of the PSP, at a significance level of 0.05. There were also no statistically significant differences in the distributions of these parameters for the measured and reconstructed parts of the PSP (Table 5).

### 3.5. Carbon Stock

The conversion of phytomass and mortmass pools to C pools was performed using data on C concentrations in the corresponding pools. These data were derived from our own experimental studies and literature (Table 6). The soil C stock was calculated separately for each subplot, given the corresponding concentration.

Validation of the data imputation technique showed that there were no significant differences in the median values of total ecosystem C stocks and C stocks in the separate pools, whether they were measured directly or calculated based on stand density (with SDT), mean H, and DRC (Table 7).

**Figure 20:**
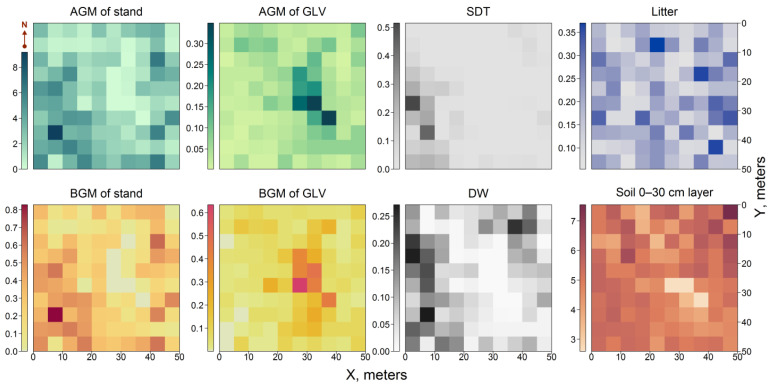
Spatial distribution of main C pools, kg m^−2^.

Data imputation enabled missing C stock values to be recovered across all 100 subplots (Figure 20).

**Figure 21:**
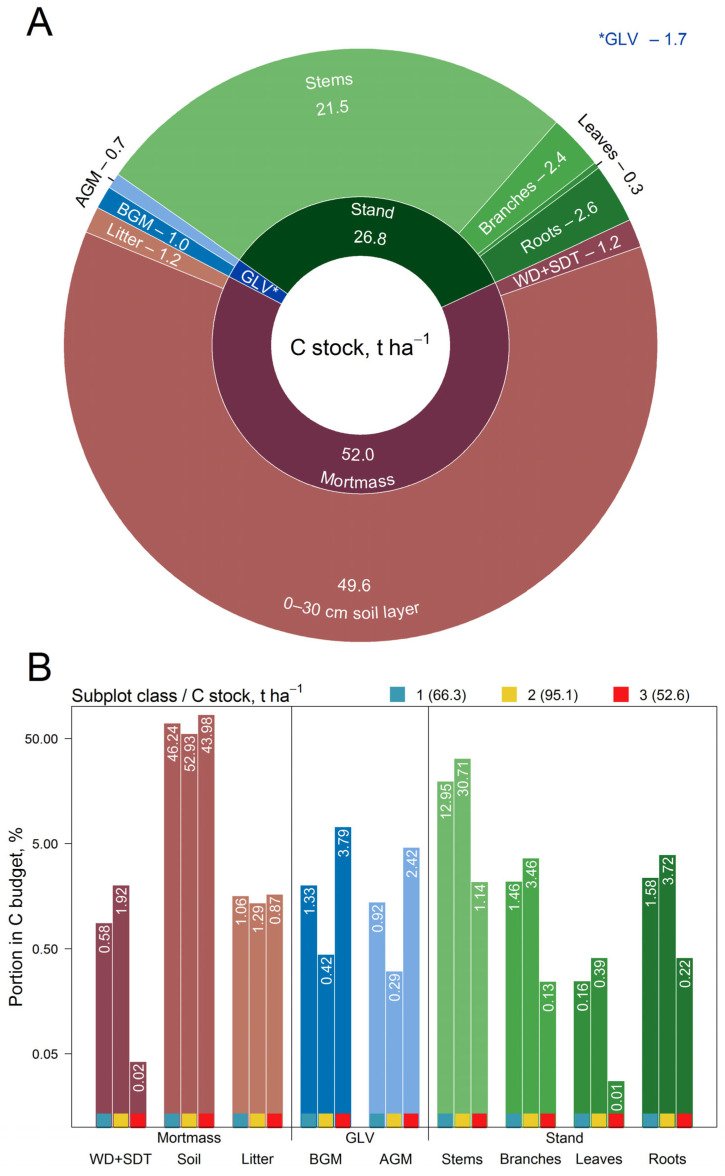
C stocks in different pools (**A**) and the contribution of subplots of different classes to the total C stock (**B**) (the y-axis is logarithmic, numbers at the tops of the bars show the absolute values of the C stocks in the corresponding pools of the corresponding classes, t ha^−1^).

The total ecosystem C stock on PSP, including the pools of living organic matter (AGM and BGM of *Betula* sp. and *S. caprea* trees, AGM and BGM of GLV), dead organic matter (litter, and the sum of SDT and WD), and the 0–30 cm soil layer, was estimated at 80.47 t ha^−1^ (Figure 21A). Living plants (trees and GLV) accumulated almost two times less C compared to dead organic matter and soil.

The C stock of subplots belonging to different classes, as well as the contribution of individual pools to the total C stock, differs significantly (Table 8). The total C stock in subplots belonging to Class 2 is significantly higher than in subplots belonging to Classes 1 and 3. However, no statistically significant differences were observed between subplots of Classes 1 and 3 (Figure 21B).

Similarly to the total C stock, the C stock in all pools of Class 2 subplots was considerably different from that of Class 1 and Class 3 subplots. C stocks in the stand, GLV, SDT, WD, litter, and soil in Class 2 subplots were higher, while C stocks in GLV were lower compared to subplots of Classes 1 and 3. Despite there being no statistically significant difference in total C stocks between subplots of Classes 1 and 3, statistically significant differences were observed in all separate pools except between litter and soil. However, the statistical significance of the difference in C stocks between the phytomass and mortmass pools in subplots of Classes 1 and 3 was lower than that observed in subplots of Classes 1 and 2, and of Classes 2 and 3.

### 3.6. Limitations and Uncertainty of Approach

The proposed method has some limitations, and the functional dependencies of C stocks on the easily measurable stand parameters obtained in this study are not universal. Firstly, the AGM of the GLV is not solely determined by stand density, but also by stand age, the species composition of the herbaceous vegetation, and edaphic conditions. We may also expect the dependence of GLV biomass on stand density to differ on sites with remarkably different climatic and/or edaphic conditions. Secondly, the soil C stock and the C:N ratio of post-agricultural ecosystems were primarily defined by soil properties at the time of agricultural land abandonment, and were relatively homogeneous within the studied area. However, the vegetation cover formed on fallow land determines the spatial differentiation of soil properties relative to the initial state. The model for SOC stock based on the C:N ratio, which was used in this research (Table 4), shows quite low explanatory power (R^2^ = 0.30). However, it has a low *p*-value (<0.0001) and a statistically significant Spearman’s correlation coefficient (0.52, *p*-value < 0.001). Increasing the spatial resolution of soil sampling would probably provide a better explanation of the variation in soil parameters and increase the reliability of the proposed model. Further studies on lands of different ages since abandonment with different edaphic conditions would help to estimate the effect of these factors on the spatial alteration of carbon accumulation. Thirdly, we did not consider the influence of microrelief, although it is clearly observable on PSP. This could indirectly affect the dynamics of soil C stocks through its influence on soil moisture conditions and tree regeneration. Comparing C stocks in subplots with different microrelief (in terms of relative elevation) revealed no significant relationships. However, we would expect differences to emerge at smaller scales (less than the subplot size of 5 × 5 m). In the current study, we primarily observed the effect of vegetation on soil C dynamics under the assumption that soil conditions at the initial stage of abandonment were uniform. However, feedback from soil to vegetation may occur, and this issue requires further chronosequence studies of the different stages of the colonization of former arable lands.

In this research, we did not consider the carbon pool accumulated in microbial biomass to be a driver of soil carbon turnover. However, soil microbiota has been shown [76,77,78,79] to be an important controlling factor in the early stages of the post-agricultural succession. Incorporating data on soil microbiota into future research will improve our understanding of the processes of C sequestration in abandoned lands and our ability to forecast.

Competition intensity in birch crowns modifies biomass allocation patterns [80]. However, the ratio of foliage biomass to stem biomass, and of foliage biomass to the biomass of stem and branches, did not differ significantly between trees subjected to different levels of competition stress. The widely accepted Optimal Partitioning Theory posits that plants allocate more biomass to organs with limited access to resources [81]. For example, in nutrient-limited soil, plants decrease the allocation of biomass to aboveground organs and increase the allocation to roots [82,83]. A similar effect has been observed in cases of reduced water availability [84,85]. Conversely, a lack of light due to intensive crown competition results in greater biomass allocation to aboveground organs [82,86]. Therefore, the biomass partitioning equations proposed in this study could overestimate foliage biomass for trees located in Class 3 subplots. However, given that only five trees are located in subplots of this Class, this calculation error can be considered insignificant.

## 4. Conclusions

The analysis revealed significant spatial variation in the total C stock and its individual components. The C stocks of the post-agricultural ecosystem, as determined on the basis of data on the spatial distribution of living trees and SDT, as well as their mean heights and diameters, do not differ significantly from the corresponding stocks calculated on the basis of direct measurements. Ecological and edaphic conditions within agricultural ecosystems are known to be relatively homogeneous due to anthropogenic influence (e.g., plowing, fertilizer application, etc.). Consequently, the structure of stocks of the main C pools revealed in this research is primarily influenced by the spatial distribution of woody and herbaceous vegetation or the amount and chemical properties of litterfall (including WD), thus enabling the suggested approach to be applied. The analysis also showed the importance of accounting for the mortmass and soil C pools, which accumulated about 2/3 of the total ecosystem stock.

The potential to divide the territory into multiple homogeneous classes, differentiated by a set of ecological and edaphic conditions, means the approach demonstrated in this study can be used to estimate C stocks in post-agricultural ecosystems. This can be achieved using both ground-based methods and remote sensing techniques.

The results of the study are important for current carbon balance estimations and predictions of the dynamics of forest ecosystems on former arable lands. The study emphasized the role of spatial heterogeneity in the ecosystem C balance and the contribution of individual C pools. The data imputation technique enables missing C stock data to be recovered on the basis of the stand characteristics. This data can be used to analyze functional relationships within post-agricultural ecosystems, simulate the dynamics of C pools, and plan reforestation and management strategies for abandoned agricultural lands.

## Figures and Tables

**Table 1:** Characteristics of measured parameters.

Measured Parameter	Scale	Number of Observations (n)	Units
DBH/DRC	individual trees	2708	cm
H	individual trees	2708	m
AGM of stand ^1^	individual trees	31	kg
Light conditions under the canopy	subplots	100	%
Cover of GLV	subplots	100	%
C and N concentrations in soil ^2^	subplots	50	%
Litter stock ^2^	subplots	50	kg m^−2^
AGM of GLV ^2^	subplots	50	kg m^−2^
BGM of GLV ^2^	reference points	9	kg m^−2^
BGM of stand ^2^	reference points	9	kg m^−2^
Bulk density of 0–30 cm soil layer ^2^	reference points	3	kg m^−3^

^1^ The parameter was measured on sample trees collected outside the PSP. ^2^ The measurements were made in 2024 after destruction of the southern part of the PSP and cover only 1250 m^2^ instead of 2500 m^2^.

**Table 2:** Stand characteristics at PSP.

Species	Status	Density, ha^−1^	DRC, mean ± SD ^1^, cm	H, mean ± SD, m
*Betula* sp.	Living	1012	5.8 ± 3.3	8.2 ± 3.3
	SDT	559	1.7 ± 1.1	3.2 ± 2.1
*S. caprea*	Living	536	3.7 ± 2.2	5.5 ± 2.0
	SDT	327	1.5 ± 0.8	3.0 ± 1.4
*P. sylvestris*	Living	70	1.6 ± 2.3	1.8 ± 1.7
	SDT	4	1.0 ± 0.0	1.1 ± 0.5

^1^ Standard deviation.

**Table 3:** Comparison of median values of the H and DRC of *Betula* sp. and *S. caprea*., located in different subplot classes on PSP using Kruskal–Wallis test and post-hoc analysis (Dunn’s test). The significance levels are as follows: * 0.05, ** 0.01, *** 0.001.

Pair	DRC	H
*Betula* sp., Classes 1 and 2	1.89	−0.02
*Betula* sp., Classes 1 and 3	1.35	2.27 *
*Betula* sp., Classes 2 and 3	1.03	2.29 *
*S. caprea*., Classes 1 and 2	3.69 **	1.82
*S. caprea*., Classes 1 and 3	2.14 *	2.23 *
*S. caprea*., Classes 2 and 3	1.31	1.84
*Betula* sp. and *S. caprea*., Class 1	5.04 ***	7.04 ***
*Betula* sp. and *S. caprea*., Class 2	11.89 ***	14.95 ***
*Betula* sp. and *S. caprea*., Class 3	1.35	1.09

**Table 4:** The equations used for parameter calculation.

Parameter	Predictor	Equation	R^2^	*p*-Value	Coeff.^1^	Value	CI ^2^ 2.5%	CI 97.5%
BGM of stand	Stand basal area	$y=a\times \left(1-e^{-b\times x}\right)$	0.96	1.01 × 10^−6^	a	2.692	1.834	4.548
					b	0.016	0.0037	0.026
BGM of GLV	Stand basal area	$y=a\times e^{-x^{b}}$	0.64	2.00 × 10^−3^	a	1.43	0.64	2.081
					b	0.59	0.29	0.81
AGM of GLV	Stand density	$y=a\times e^{-b\times \sqrt{x}}$	0.75	1.25 × 10^−7^	a	0.83	0.68	0.89
					b	4.01	3.37	5.048
C:N ratio in 0–30 cm soil layer	Stand density (with SDT)	y=a+log(1+x×b)	0.57	2.51 × 10^−10^	a	8.71	0.68	0.89
					b	17.89	3.37	5.048
Soil C concentration	C:N ratio in 0–30 cm soil layer	$y={\log\left(x-a\right)}^{b}$	0.30	3.75 × 10^−5^	a	7.52	6.95	8.53
					b	0.69	0.52	0.81
WD mass	AGM of GLV	$y=e^{\left(-a\times \sqrt{x}\right)}$	0.59	1.94 × 10^−6^	a	7.96	6.84	9.31
*Betula* sp. stem mass portion	DRC	$y=\frac{a}{1+b\times e^{-c\times x}}$	0.98	2.33 × 10^−7^	a	0.89	0.87	0.91
					b	2.030	1.90	2.44
					c	1.34	0.96	1.86

^1^ The letters (a, b, c) denote equation coefficients (refer to the “Equation” column). ^2^ Confidence interval.

**Table 5:** A comparison of the mean values and distributions of the parameters calculated during the data imputation for the measured and reconstructed parts of the PSP.

		AGM of GLV	C:N Ratio in 0–30 cm Soil Layer	Soil C Concentration	WD Mass
Wilcoxon’s test	W	1388	1197	1565	991
	*p*-value	0.34	0.72	0.31	0.095
Kolmogorov–Smirnov test	D	0.20	0.12	0.24	0.22
	*p*-value	0.25	0.85	0.14	0.18

**Table 6:** C concentration (%) in different biomass and mortmass pools.

Pool	Species	Organ	C, %	Source
Stand phytomass	*Betula* sp.	Stem	50.37	Own measurements
		Branches	51.89	Own measurements
		Leaves	52.88	Own measurements
		Roots	50.20	[67]
	*S. caprea*	Stem	49.80	[46,47,68]
		Branches	49.00	
		Leaves	46.40	
		Roots	46.30	
GLV	*S. canadensis*	-	44.66	[69]
	*C. epigejos*	-	43.25	[70]
	*A. podagraria*	-	38.24	[71]
	*C. sylvatica*	-	44.78	[72]
Litter ^1^	*S. canadensis*	-	24.10	Calculated from [73]
	*C. epigejos*	-	30.28	Calculated from [74]
SDT/WD	*Betula* sp., *S. caprea*	Stem, branches	50.00	[75]
Soil (0–30 cm)	-	-	0.74–1.73	Own measurements

^1^ The litter pool was mainly composed of aboveground litter from *S. canadensis* and *C. epigejos*, as well as woody debris. Other plant species and fast-decomposing fractions of tree litter were almost entirely absent from the samples. Therefore, no parameters could be determined for these components.

**Table 7:** Comparison of median values of C stocks, calculated directly from field measurements of the corresponding pools (“obs”), and those calculated from the stand density (with SDT), mean DRC, and H (“mod”).

	Total	Mortmass and Soil			GLV		Stand			
		Litter	DW+SDT	Soil	AGM	BGM	Stems	Branches	Leaves	Roots
mod	7.648	0.102	0.133	5.356	0.057	0.046	1.339	0.149	0.017	0.219
obs	7.578	0.103	0.115	5.293	0.059	0.051	1.391	0.156	0.017	0.219
*p*-value	0.854	0.813	0.573	0.588	0.768	0.727	0.797	0.758	0.747	0.938

**Table 8:** Comparison of the median values of the main components of the ecosystem C stock on PSP using Kruskal–Wallis test and post-hoc analysis (Dunn’s test). The significance levels are as follows: * 0.05, ** 0.01, *** 0.001.

Pool of C	Classes 1 and 2	Classes 1 and 3	Classes 2 and 3
Total	−6.49 ***	1.49	4.63 ***
WD+SDT	−6.11 ***	2.23 *	5.20 ***
Soil	−4.70 ***	0.29	2.55 **
Litter	−2.36 **	1.07	2.22 *
AGM of GLV	6.38 ***	−2.02 *	−5.11 ***
BGM of GLV	6.67 ***	−2.00 *	−5.23 ***
Tree stems	−5.70 ***	2.25 *	5.02 ***
Tree branches	−5.70 ***	2.25 *	5.02 ***
Tree leaves	−5.71 ***	2.25 *	5.03 ***
Tree roots	−6.14 ***	2.00 *	4.98 ***

## Data Availability

The data are available from the authors upon reasonable request.

## Supplementary Materials

The following supporting information can be downloaded at: https://www.mdpi.com/article/10.3390/plants14152401/s1, Figure S1: The fragment of the PSP corresponding to subplots of Class 1; Figure S2: The fragment of the PSP corresponding to subplots of Class 2; Figure S3: The fragment of the PSP corresponding to subplots of Class 3.

## Abbreviations

The following abbreviations are used in this manuscript:

ECS	Ecological-climatic station
PSP	Permanent sampling plot
SDT	Standing dead trees
GLV	Ground layer vegetation
WD	Woody debris
AGM	Aboveground phytomass
BGM	Belowground phytomass
DBH	Diameter at breast height
DRC	Diameter at root collar
H	Height
C	Carbon
N	Nitrogen
GLI	Global light index
