# Supplementary figures and images for: Spatial Heterogeneity in Carbon Pools of Young Betula sp. Stands on Former Arable Lands in the South of the Moscow Region
Source: Plants (Basel). 2025 Aug 3;14(15):2401. doi: 10.3390/plants14152401 (PMC12349199; doi:10.3390/plants14152401)

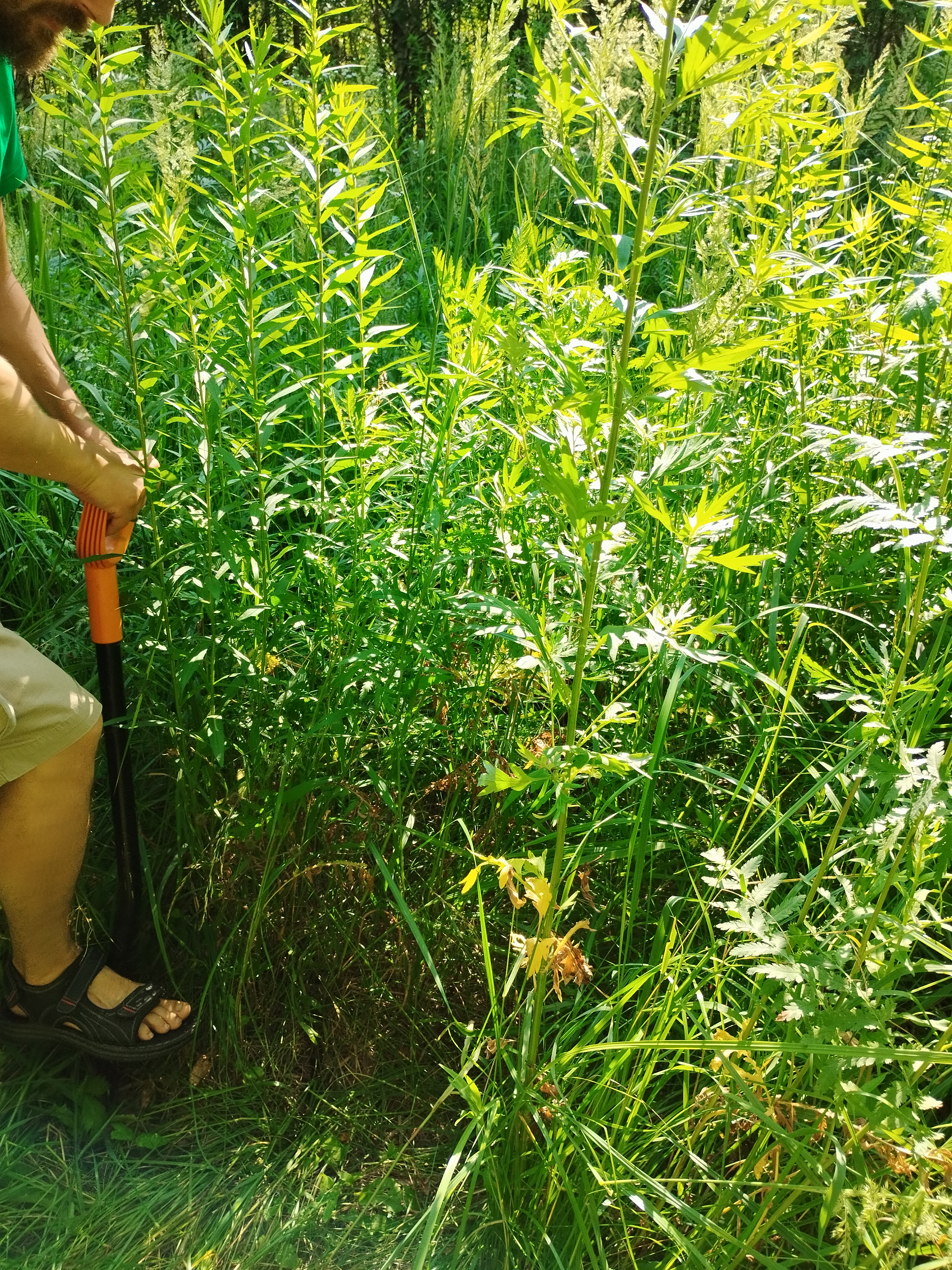

Supplement: Supplementary file 1 [file plants-14-02401-s001.zip › Fig_S3.jpg]

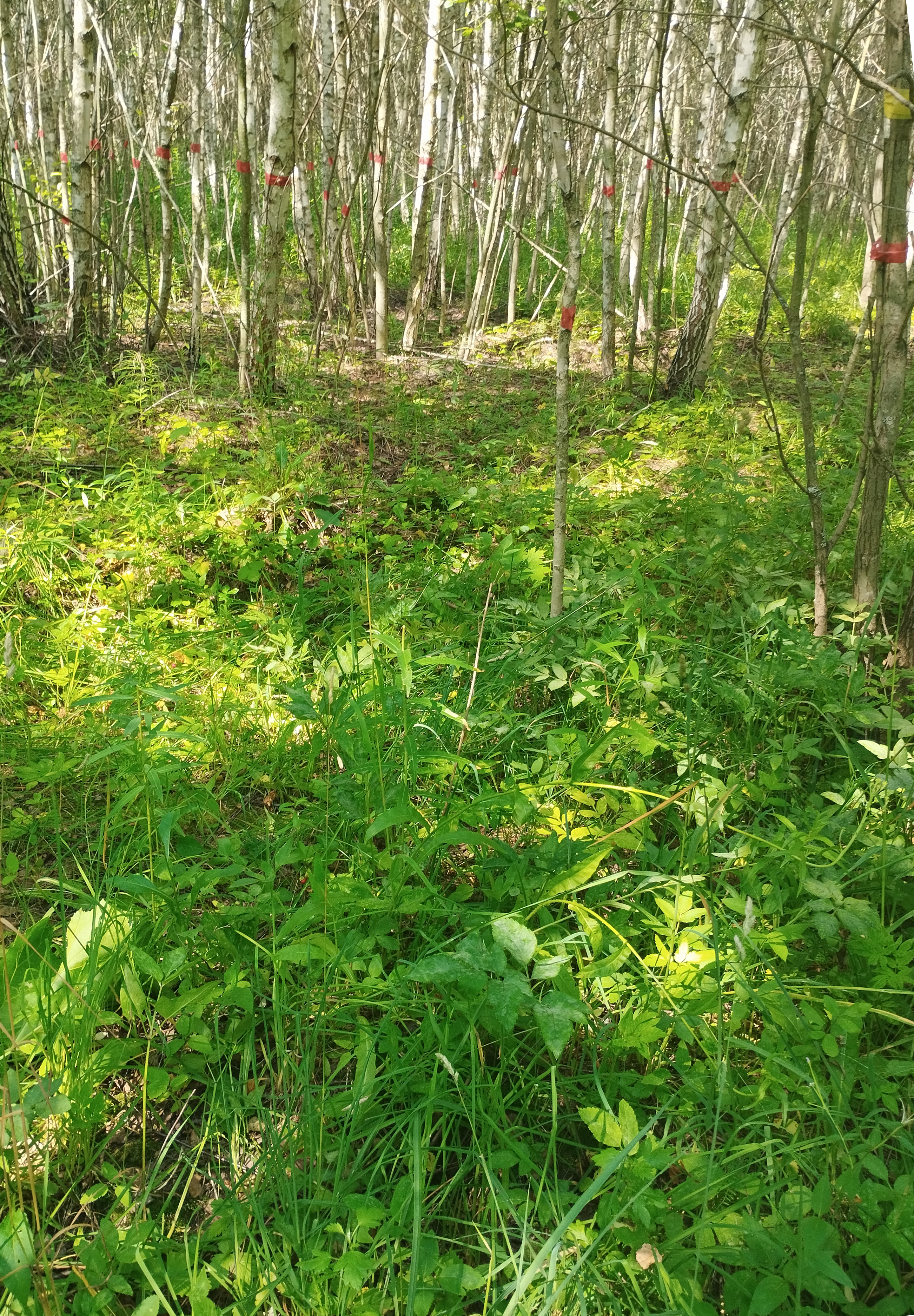

Supplement: Supplementary file 1 [file plants-14-02401-s001.zip › Fig_S1.jpg]

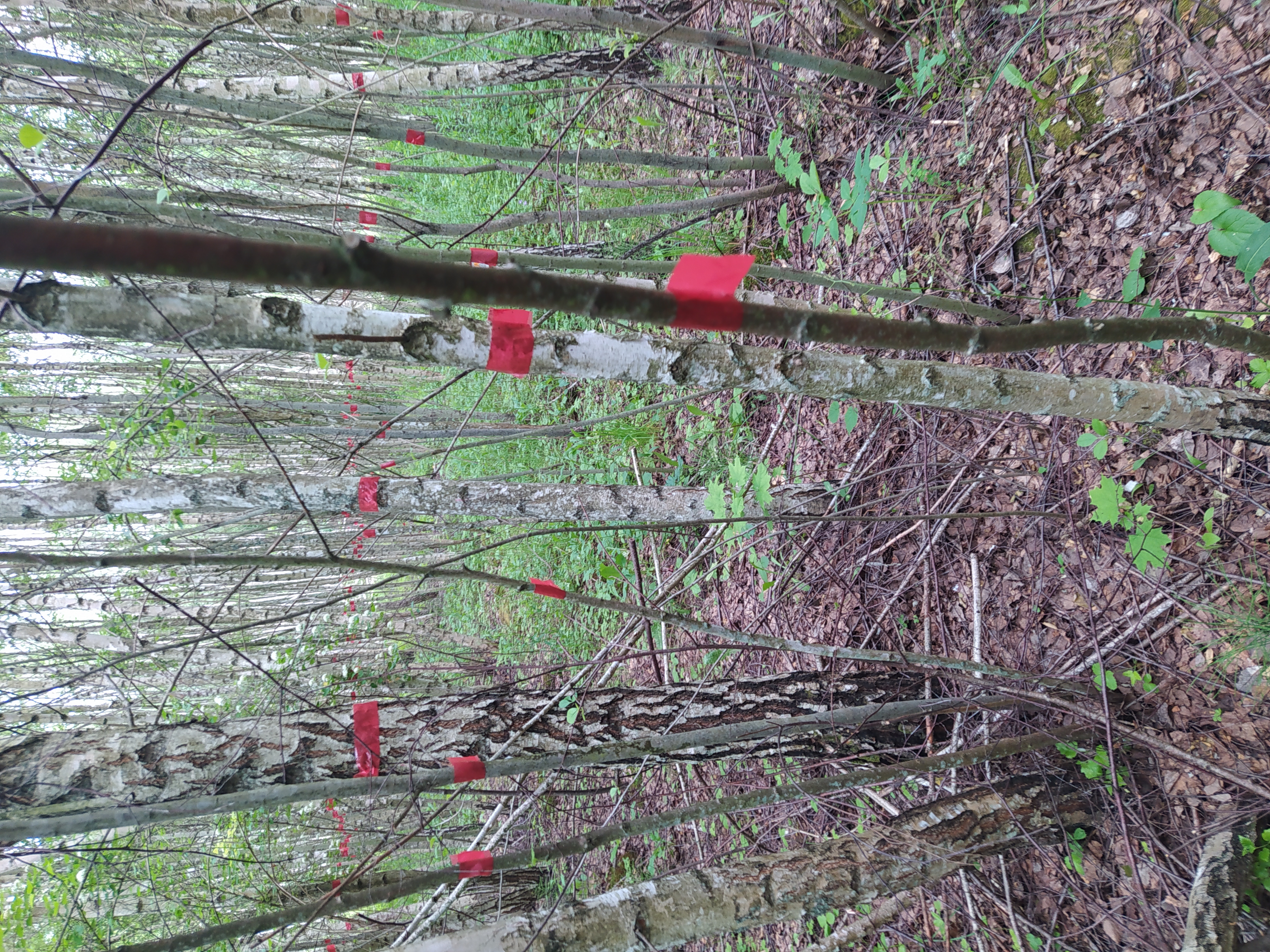

Supplement: Supplementary file 1 [file plants-14-02401-s001.zip › Fig_S2.jpg]
